# Cerebroplacental Ratio as a Predictor of Perinatal Outcome in Hypertensive Disorders of Pregnancy and Its Comparison With Its Constituent Doppler Indices

**DOI:** 10.7759/cureus.49951

**Published:** 2023-12-05

**Authors:** Neeru Malik, Sandhya Jain, Rajiv Ranjan, Divya Maurya, Nikita Madan, Uday K Singh, Vinayak Malik, Sanjay Choudhary, Anupa Singhal, Natasha Tyagi

**Affiliations:** 1 Obstetrics and Gynecology, Dr. Baba Saheb Ambedkar Medical College & Hospital, Delhi, IND; 2 Radiology, Dr. Baba Saheb Ambedkar Medical College & Hospital, Delhi, IND; 3 Obstetrics and Gynecology, Employees' State Insurance Corporation (ESIC) Hospital & Post-Graduate Institute of Medical Science & Research (PGIMSR), Delhi, IND; 4 Computer Science, University of Wisconsin, Madison, USA; 5 Pediatrics and Neonatology, Dr. Baba Saheb Ambedkar Medical College & Hospital, Delhi, IND

**Keywords:** perinatal outcome, middle cerebral artery, umbilical artery, cerebroplacental ratio, doppler ultrasound

## Abstract

Objective

Doppler velocimetry is an established method of antepartum fetal surveillance in pre-eclampsia. Cerebroplacental ratio detects the centralization of fetal blood flow and the insufficiency in placental circulation. It is postulated to be a better marker of perinatal outcome than either vessel Doppler alone. The current study aims to assess the cerebroplacental ratio as a predictor of adverse perinatal outcomes and compare it to the systolic/diastolic (S/D) ratio of umbilical artery (UA) and middle cerebral artery (MCA) in hypertensive disorders of pregnancy.

Material and methods

The present prospective observational cohort study included 100 patients with hypertensive disorders of pregnancies between 32 and 37 weeks. Ultrasound with Doppler was done and the following parameters were assessed: fetal biometry, amniotic fluid index, umbilical artery pulsatility index, middle cerebral artery pulsatility index, S/D ratio of umbilical artery, S/D ratio of middle cerebral artery, and cerebroplacental ratio. Sensitivity, specificity, positive and negative predictive values were calculated for the cerebroplacental ratio and S/D ratios of umbilical and middle cerebral arteries. McNemar’s test was used for the comparison of sensitivity and specificity.

Results

Thirty-two patients had an abnormal cerebroplacental ratio. Adverse perinatal outcomes such as a cesarean section for fetal distress, small for gestational age, APGAR < 7 at 1 and 5 minutes, NICU admission, and perinatal mortality were more in the group with abnormal cerebraplacental ratio and the difference was statistically significant.

Conclusion

The cerebroplacental ratio is a more reliable predictor of adverse perinatal outcomes and should be routinely calculated during obstetrical Doppler for antepartum fetal surveillance in case of hypertensive disorders of pregnancy. It suggested that the cerebroplacental ratio may be calibrated in the software of the Doppler ultrasonography machine for routine use in high-risk pregnancies.

## Introduction

The hypertensive disorders of pregnancy (HDP) complicate about 3-10% of pregnancies [1]. They are one of the major contributors to maternal and fetal mortality and morbidity globally, with approximately 30,000 maternal and 500,000 perinatal deaths attributed to the HDP annually [2-4].

Doppler ultrasound velocimetry of uteroplacental umbilical and fetal vessels has become an established method of antenatal fetal surveillance which allows a noninvasive assessment of fetal circulation. Doppler indices reflect important information on the hemodynamics of the vascular area under study. Umbilical arteries (UA) are the most common vessels assessed by Doppler ultrasound. The umbilical arterial circulation is normally a low-impedance circulation, it reflects the status of the placental circulation, and the increase in end-diastolic flow that is seen with advancing gestation is a direct result of an increase in the number of tertiary stem villi that takes place with placental maturation. The middle cerebral artery (MCA) is the most accessible cerebral vessel to ultrasound Doppler imaging in the fetus and it carries more than 80% of cerebral blood flow [5].

The cerebral circulation is normally a high-impedance circulation with continuous forward flow throughout the cardiac cycle. In the presence of fetal hypoxemia, central redistribution of blood flow happens, resulting in increased blood flow to the brain, heart, and adrenals and a decreased flow to the peripheral and placental circulations. This blood flow redistribution is known as the brain-sparing effect and plays a major role in fetal adaptation to oxygen deprivation [6].

The cerebroplacental ratio (CP ratio), the ratio of the pulsatility index (PI) of the MCA to that of the UA, can detect fetal hypoxemia occurring via two different mechanisms: reduced resistance in the MCA (brain-sparing effect) and increasing placental resistance as evidenced by a decrease in umbilical artery PI [7].

Recent studies have shown that combining these two parameters in the CP ratio further improves the prediction as it reflects not only the circulatory insufficiency of the umbilical vessels of the placenta, manifested by alterations in the umbilical artery parameters but also the adaptive changes resulting in modifications of the middle cerebral artery parameters [8]. So, the CP ratio is postulated to be a better predictor of adverse perinatal outcomes, with higher diagnostic accuracy, sensitivity, and positive predictive value than either vessel Doppler indices considered alone [9].

The CP ratio is considered to be a marker of centralization of fetal blood flow as an adaptation to placental insufficiencies, such as that seen in pre-eclampsia [10]. The CP ratio is correlated with adverse perinatal outcomes and impaired long-term neonatal cognitive development [11].

Therefore, the present study has been undertaken to evaluate the efficacy of the CP ratio as a predictor of perinatal outcome in comparison to Doppler indices of umbilical and middle cerebral arteries in the hypertensive disorders of pregnancy.

## Materials and methods

Study design and setting

A two-year prospective observational study was conducted at a tertiary care facility in Delhi (Dr. Baba Saheb Ambedkar Hospital).

The study was approved by the Institutional Ethics and Research Board of the Dr. Baba Saheb Ambedkar Medical College with approval number F5(59)2017/BSAH/DNB/29973 dated November 28, 2019. Informed consent was obtained from each patient in their vernacular language before enrollment.

Sample size

In a previous study, the sensitivity and specificity of the CP ratio for predicting adverse perinatal outcomes were 94.42% and 82.65% respectively. Taking these values as a reference, the minimum required sample size with a desired precision of 10%, 80% power of study, and 95% level of significance was 93 patients. To reduce the margin of error, the total sample size was 100 [12].

Inclusion and exclusion criteria

Women with hypertension peculiar to pregnancy (pre-eclampsia, eclampsia, and gestational hypertension) are diagnosed according to the American Congress of Obstetricians and Gynecologists (ACOG) guidelines (2019) with a gestational age between 32 to 37 weeks. singleton pregnancy, live fetus, cephalic presentation, and intact amniotic membranes were included in the study [13].

Patients with multiple pregnancies, anomalous fetuses, antepartum hemorrhage, intrauterine fetal demise, diabetes mellitus, severe anemia, Rh-negative pregnancy, and chronic hypertension were excluded from the study.

Methodology

Pregnant women attending antenatal OPD, admitted in the antenatal ward and the labor room of the department of obstetrics and gynecology with the fulfillment of inclusion and exclusion criteria were recruited for the study. Written informed consent was taken from all eligible patients. Detailed history was taken and a complete general, systemic, and obstetric examination was performed. Gestational age was determined by the date of the last menstrual period or by the first-trimester ultrasound. All relevant investigations were carried out. The relevant data was recorded in the proforma.

All guidelines of the Pre-Conception and Pre-Natal Diagnostic Techniques (PCPNDT) Act, 1994 were followed while conducting ultrasound/Doppler, and the following parameters were assessed: fetal biometry, amniotic fluid index (AFI), mean UA PI, mean MCA PI, S/D ratio of UA, S/D ratio of MCA, and CP ratio.

The sonographic assessment was done in recruited patients using a Samsung Medison (EVN4-9) ultrasound machine using a 3.5 MHz transabdominal probe. Umbilical Doppler waveforms were obtained from a free-floating loop of the umbilical cord during minimal fetal activity. Three similar consecutive waveforms were obtained, mean PI of these waveforms was used for UA PI analysis. For MCA, color Doppler was used to identify the circle of Willis in a magnified axial plane of the fetal head obtained at the base of the skull, at the level of the thalami and wings of the sphenoid bone. In this plane, proximal and distal middle cerebral arteries are seen in a long axis with their course almost parallel to the ultrasound beam, the insonating beam, which is parallel to the vessel and has an angle of insonation of 0 degrees. Pulsed-wave Doppler gate was positioned in the proximal third portion of the vessel, close to its origin from the circle of Willis, velocity measurement was optimal when the insonating angle was as close to zero as possible. Angle correction is performed if 0-10 degrees. Three similar consecutive waveforms were obtained. The mean PI of these waveforms was used for MCA PI analysis.

The UA PI was considered abnormal when the values were > 95th percentile. MCA PI is considered abnormal when the values are < 5th percentile of previously published values for gestational age [14]. MCA S/D ratio < 3 was considered abnormal and S/D ratio of umbilical artery >3 was considered abnormal. [15] The CP ratio was obtained using the following ratio: mean MCA PI/mean UA PI.

As in some previous studies, a single cutoff value (1.08) was used in the present study, above which velocimetry was considered normal and below which was considered abnormal [15,16].

The study was non-blinded as both the obstetrician and the patient were aware of the result of the scan. Patients with normal Doppler were followed every 2 weeks. Patients with abnormal Doppler were followed biweekly. The fetal surveillance was continued until the decision of delivery was taken. The last ultrasound and Doppler indices before delivery were considered in the study. The patients were managed as per institutional protocol. The patients were followed till delivery and the following adverse perinatal outcomes were recorded.

The composite adverse perinatal outcome was defined as the presence of any one of the following adverse outcomes in the study population: (1) cesarean section for fetal distress, (2) instrumental delivery for fetal distress, (3) small for gestation age (birth weight less than 10th percentile for gestational age), (4) meconium stained liquor (MSL), (5) APGAR score < 7 at 1 and 5 minutes, (6) admission to the neonatal intensive care unit (NICU), and (7) stillbirth/perinatal death.

The composite adverse perinatal outcome was calculated for comparing the predictive value of the CP ratio with other Doppler indices (S/D ratio of UA and MCA) in the prediction of adverse outcomes in hypertensive disorders of pregnancy.

Statistical analysis

The categorical variables were presented as numbers and percentages (%). On the other hand, the quantitative data were presented as the mean ± standard deviation (SD) and as the median with 25th and 75th percentiles (interquartile range). The comparison of the qualitative variables was analyzed using the chi-square test. If any cell had an expected value less than 5, then Fisher’s exact test was used. Sensitivity, specificity, positive predictive value (PPV), and negative predictive value (NPV) of CP ratio and S/D ratios of umbilical and middle cerebral arteries were calculated. McNemar’s test was used for the comparison of sensitivity and specificity. The p-value less than 0.05 was considered statistically significant.

## Results

Maternal characteristics, Doppler findings, and perinatal outcomes of study subjects are shown in Table 1. Sixty percent of the patients were primigravida and 71% of the patients were nulliparous. These two factors showed remarkable association with hypertensive disorders of pregnancy as expected. The mean age was 26 years. 

**Table 1:** Maternal characteristics, Doppler findings, and perinatal outcome of study subjects. UA: umbilical artery; MCA: middle cerebral artery; MSL: meconium stained liquor; NICU: neonatal intensive care unit.

Patient’s details	Frequency	Percentage
Age (years)		
20-25	46	46.00%
26-30	35	35.00%
31-35	19	19.00%
Mean ± SD	26.26 ± 3.8	
Gravidity		
Primi	60	60.00%
Multi	40	40.00%
Parity		
Nulliparity	71	71.00%
Multiparity	29	29.00%
Booked/unbooked		
Booked	81	81.00%
Unbooked	19	19.00%
Gestational age at delivery (weeks)		
< 37	74	74.00%
≥ 37	26	26.00%
Mean ± SD	36.19 ± 1.01	
Hypertensive disorder of pregnancy	Frequency	Percentage
Gestational hypertension	29	29.00%
Pre-eclampsia	42	42.00%
Severe preeclampsia	21	21.00%
Eclampsia	8	8.00%
Cerebroplacental ratio and other Doppler indices		
S/D ratio (UA) {>3 abnormal}	15	15%
S/D ratio (MCA) {<3 abnormal}	16	16.00%
Cerebroplacental ratio {<1.08 abnormal}	32	32.00%
Adverse perinatal outcome	Frequency (n=58)	Percentage
Cesarean section for fetal distress	44	75.86%
Vaginal instrumental delivery for fetal distress	3	5.17%
Small for gestational age	28	48.28%
APGAR at 1 minute (<7)	28	48.28%
APGAR at 5 minutes (<7)	15	25.86%
MSL	30	51.72%
NICU admission	28	48.28%
Stillbirth/Perinatal death	4	6.90%

The perinatal outcomes which showed significant association with abnormal CP ratio such as cesarean section for fetal distress 28 (87.50%), small for gestational age (SGA) babies 24 (75%), NICU admission 22 (68.75%), APGAR scores < 7 at 1 minute 22 (68.75%), APGAR scores < 7 at 5 minutes 12 (37.50%) and stillbirth/perinatal death 4 (12.50%). The association of operative vaginal delivery for fetal distress 0 (0%) and MSL 12 (37.50%) with abnormal CP ratio was statistically not significant (Table 2).

**Table 2:** Association of adverse perinatal outcome with cerebroplacental ratio (<1.08 abnormal). *Significant at p<0.05; **Chi-squared test; ***Fisher's exact test. MSL: meconium stained liquor; NICU: neonatal intensive care unit.

Outcomes	Normal (n=68)	Abnormal (n=32)	Total	p-value
Cesarean section for fetal distress*	16 (23.53%)	28 (87.50%)	44 (44%)	< 0.0001**
Vaginal instrumental delivery	3 (4.41%)	0 (0%)	3 (3%)	0.549***
Small for gestational age*	4 (5.88%)	24 (75%)	28 (28%)	< 0.0001**
APGAR < 7 at 1 minute*	6 (8.82%)	22 (68.75%)	28 (28%)	< 0.0001**
APGAR < 7 at 5 minutes*	3 (4.41%)	12 (37.50%)	15 (15%)	< 0.0001**
MSL	18 (26.47%)	12 (37.50%)	30 (30%)	0.262**
NICU admission*	6 (8.82%)	22 (68.75%)	28 (28%)	< 0.0001**
Stillbirth/ perinatal death*	0 (0%)	4 (12.50%)	4 (4%)	0.009***

As shown in Table 3, out of 100 women, 58 had adverse outcomes. Among these 32 (100%) had an abnormal CP ratio, whereas 26 (38.24%) women who had adverse pregnancy outcomes had a normal CP ratio. Fifteen women had an abnormal S/D ratio of the umbilical artery; among these 15 (100%) had an adverse outcome. However, 43 (50.59%) women who had adverse pregnancy outcomes had a normal S/D ratio of umbilical artery. Sixteen women had an abnormal S/D ratio of the middle cerebral artery; among these 9 (56.25%) had an adverse perinatal outcome (p < 0.877).

**Table 3:** Association of adverse perinatal outcome with various Doppler indices. CP ratio: cerebroplacental ratio; S/D ratio: systolic/diastolic ratio; UA: umbilical artery; MCA: and middle cerebral artery.

N (%)	CP ratio		S/D ratio (UA)		S/D ratio (MCA)	
	Normal (n=68)	Abnormal (n=32)	Normal (n=85)	Abnormal (n=15)	Normal (n=84)	Abnormal (n=16)
Adverse perinatal outcome	26 (38.24%)	32 (100%)	43 (50.59%)	15 (100%)	49 (58.33%)	9 (56.25%)
p-value	< 0.001		< 0.001		0.877	

As seen in Table 4, the CP ratio had better sensitivity (55.17%), specificity (100%), PPV (100%), and NPV (61.76%) for predicting adverse perinatal outcomes as compared to other Doppler indices (S/D ratio of the UA and MCA with p = 0.001 and p = 0.0001 respectively).

**Table 4:** Comparison of result of cerebroplacental ratio with other Doppler indices in prediction of composite adverse perinatal outcome. * significant at p less than 0.05; ** McNemar's test. CP ratio: cerebroplacental ratio; PPV: positive predictive value; NPV: negative predictive value; S/D ratio: systolic/diastolic ratio; UA: umbilical artery; MCA: middle cerebral artery.

Predictive values	Cerebroplacental ratio	S/D ratio (UA)	S/D ratio (MCA)
Sensitivity (95% CI)	55.17% (41.54% to 68.26%)	25.86% (15.26% to 39.04%)	15.52% (7.35% to 27.42%)
Specificity (95% CI)	100% (91.59% to 100%)	100% (91.59% to 100%)	83.33% (68.64% to 93.03%)
Positive Predictive Value (95% CI)	100% (89.11% to 100%)	100% (78.20% to 100%)	56.25% (29.88% to 80.25%)
Negative Predictive Value (95% CI)	61.76% (49.18% to 73.29%)	49.41% (38.39% to 60.48%)	41.67% (31.00% to 52.94%)
Diagnostic accuracy	74.00%	57.00%	44.00%
P-value comparing sensitivity with CP ratio*	-	0.001**	0.0001**
P-value comparing specificity with CP ratio*	-	-	0.016**

## Discussion

Hypertension peculiar to pregnancy (pre-eclampsia, gestational hypertension, and eclampsia) is characterized by reduced organ perfusion secondary to vasospasm, ischemia, and endothelial dysfunction and leads to adverse perinatal outcomes such as fetal growth restriction, low birth weight, spontaneous or iatrogenic preterm delivery, stillbirth, respiratory distress syndrome, and admission to neonatal intensive care [17].

In the present study, the association of cesarean section for fetal distress with abnormal CP ratio was 87.50%. This is comparable to the findings in a study by Sahinaj et al. which showed that the correlation between cesarean section for fetal distress and CP ratio was 71.9% [18].

The present study showed a statistically significant correlation between SGA and abnormal CP ratio (75%) with sensitivity, specificity, NPV, and diagnostic accuracy of 85.71%, 88.89%, 94.12%, and 88% respectively. The current study reported a statistically significant correlation between the CP ratio and APGAR < 7 at 5 minutes with sensitivity, specificity, PPV, NPV, and diagnostic accuracy of 80%, 76.47%, 37.5%, 95.59%, and 77% respectively. Our results agreed with the study by Alanwar et al., who reported CP ratio had excellent prognostic accuracy of APGAR at <7 AT 5 minutes with 50% sensitivity, 88.1% specificity, and 82% accuracy respectively [19]. Also, Sahinaj et al. reported a significant positive correlation between abnormal CP ratio and APGAR at < 7 at 5 minutes (61.9%) with 62.8% sensitivity and 76.8% specificity respectively [18].

The present study observed a statistically significant correlation between abnormal CP ratio and NICU admission (68.75%) with sensitivity of 78.57%, specificity of 86.11%, PPV of 68.75%, and NPV of 91.18%. This is similar to a study done by Alanwar et al. who reported a significant correlation between CP ratio and NICU admission with 62.5% sensitivity, 71.43% specificity, 90.9% NPV, and 70% accuracy respectively [19]. Also, Sahinaj et al. reported a significant positive correlation between abnormal CP ratio and NICU admission with sensitivity, specificity, PPV, and NPV as 50.1%, 79.3%, 77.6%, and 52.5% respectively [18]. Similarly, a previous study from India reported that CPR had a high sensitivity (93.3% ), specificity (74.47%), PPV (53.85% ), and NPV (97.22%) for prediction of NICU admission in hypertensive disorders of pregnancy [20]. Table 5 summarizes the comparison of the sensitivity, specificity, PPV, and NPV of cerebroplacental ratio for NICU admission among various previous studies. Regan et al. in a retrospective study on 270 women found Cerebroplacental to be significantly associated with subsequent development of pre-eclampsia in pregnancies complicated by suspected fetal growth restriction (FGR). They reported abnormal CP ratio to be associated with earlier gestation delivery and a higher rate of neonatal admission as compared to abnormal isolated UA Doppler with normal CP ratio and also showed high perinatal mortality in the abnormal CP ratio group. Adverse perinatal outcome (MSL, APGAR <7 at 5 minutes, arterial cord pH < 7.10, NICU admission, perinatal mortality) was higher in in abnormal CP ratio group (85%) as compared to the normal CP ratio with abnormal UA Doppler group (36.5%) [21].

**Table 5:** Comparison of the predictive value of cerebroplacental ratio for NICU admission among various previous studies. * significant at <0.05; ** McNemar's test. PPV: positive predictive value; NPV negative predictive value; DA: diagnostic accuracy; NICU: neonatal intensive care unit.

Author	Sensitivity	Specificity	PPV	NPV	DA	p-value
Sahinaj et al. [18]*	50.1%	79.3%	77.6%	52.5%	-	<0.0001**
El Guindy et al. [22]	41.6%	85%	77%	54.5%	61.2%	-
Alanwar et al. [19]*	62.5%	71.43%	29.4%	90.9%	70%	0.009**
Sharma et al. [23]*	42%	70.21%	60%	53.23%	55.67%	0.001**
Nayak et al. [20]	93.3%	74.47%	53.85%	97.22%	-	-
Zarean et al. [24]	37%	63%	27%	7%	56%	-
Present Study*	78.57%	86.11%	68.75%	91.18%	84.00%	<0.0001**

The current study showed a statistically significant correlation between abnormal CP ratio and stillbirth/perinatal death, estimated as 12.50 % with 100 % sensitivity, 70.83 % specificity, 12.50% PPV, and 100% NPV. Similar results were reported by Sahinaj et al., who observed a significant positive correlation between abnormal Cp ratio and perinatal death with sensitivity, specificity, PPV, and NPV as 98%, 66%, 30.8%, and 99.7% respectively [18].

In the present study, the predictive value of the CP ratio for composite adverse perinatal outcomes demonstrated high specificity and PPV of 100% each with a sensitivity of 55.17% and a diagnostic accuracy of 74%. This demonstrates that the CP ratio has a significant correlation in predicting adverse perinatal outcomes. CP ratio had better sensitivity (55.17%), specificity (100%), PPV (100%), and NPV (61.76%) for predicting adverse perinatal outcomes as compared to other Doppler indices (S/D ratio of the UA and MCA with p = 0.001 and p = 0.0001 respectively). In a comparative evaluation of diagnostic accuracy, the CP ratio had better diagnostic accuracy (74%) than isolated Doppler indices [MCA (44%), UA (57%)] to predict adverse perinatal outcomes in hypertensive disorders of pregnancies. In the study by Smitha et al., women with pregnancy-induced hypertension between 28-40 weeks of gestation were studied for umbilical artery and middle cerebral artery Doppler waveform [12]. They reported Cerebroplacental ratio (MCA PI/UA PI) had the highest sensitivity (94.42%), PPV (86.42%), and accuracy (90%) for predicting adverse perinatal outcomes than UA PI and MCA PI [12].

## Conclusions

Hypertensive disorders of pregnancy are associated with significant perinatal morbidity and mortality. The cerebroplacental (CP) ratio is a better predictor of adverse perinatal outcomes with high diagnostic accuracy, sensitivity, positive predictive value, and negative predictive value than either vessel Doppler indices considered alone in hypertensive disorders of pregnancy. It is suggested that the CP ratio may be calibrated in the software of the Doppler ultrasound machine for routine use in antepartum fetal surveillance of high-risk pregnancies.
